# 5-Bromo-3-(4-chloro­phenyl­sulfin­yl)-2-methyl-1-benzofuran

**DOI:** 10.1107/S1600536810038870

**Published:** 2010-10-09

**Authors:** Hong Dae Choi, Pil Ja Seo, Byeng Wha Son, Uk Lee

**Affiliations:** a Department of Chemistry, Dongeui University, San 24 Kaya-dong Busanjin-gu, Busan 614-714, Republic of Korea; b Department of Chemistry, Pukyong National University, 599-1 Daeyeon 3-dong, Nam-gu, Busan 608-737, Republic of Korea

## Abstract

In the title compound, C_15_H_10_BrClO_2_S, the 4-chloro­phenyl ring is oriented approximately perpendicular to the mean plane of the benzofuran ring [dihedral angle = 89.55 (9)°]. In the crystal, mol­ecules are linked through weak inter­molecular C—H⋯O hydrogen bonds and and a Br⋯Br contact [3.783 (3) Å].

## Related literature

For the biological activity of benzofuran compounds, see: Aslam *et al.* (2006 ▶); Galal *et al.* (2009 ▶); Khan *et al.* (2005 ▶). For natural products with benzofuran rings, see: Akgul & Anil (2003 ▶); Soekamto *et al.* (2003 ▶). For the structures of related 3-(4-chloro­phenyl­sulfin­yl)-2-methyl-1-benzofuran derivatives, see: Choi *et al.* (2010**a* ▶,b* ▶).
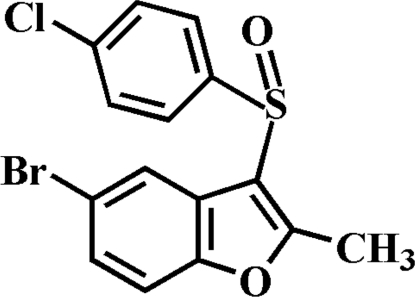


## Experimental

### 

#### Crystal data

C_15_H_10_BrClO_2_S
*M* *_r_* = 369.65
Monoclinic, 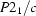

*a* = 11.530 (6) Å
*b* = 5.834 (3) Å
*c* = 22.045 (13) Å
β = 100.602 (16)°
*V* = 1457.6 (15) Å^3^
*Z* = 4
Mo *K*α radiation
μ = 3.14 mm^−1^
*T* = 173 K
0.20 × 0.16 × 0.15 mm

#### Data collection

Bruker SMART APEXII CCD diffractometer
Absorption correction: multi-scan (*SADABS*; Bruker, 2009 ▶) *T*_min_ = 0.625, *T*_max_ = 0.746
12344 measured reflections
3169 independent reflections
2505 reflections with *I* > 2σ(*I*)
*R*_int_ = 0.054

#### Refinement

*R*[*F* ^2^ > 2σ(*F* ^2^)] = 0.040
*wR*(*F* ^2^) = 0.125
*S* = 1.08
3169 reflections
182 parameters
H-atom parameters constrained
Δρ_max_ = 0.37 e Å^−3^
Δρ_min_ = −0.61 e Å^−3^

### 

Data collection: *APEX2* (Bruker, 2009 ▶); cell refinement: *SAINT* (Bruker, 2009 ▶); data reduction: *SAINT*; program(s) used to solve structure: *SHELXS97* (Sheldrick, 2008 ▶); program(s) used to refine structure: *SHELXL97* (Sheldrick, 2008 ▶); molecular graphics: *ORTEP-3* (Farrugia, 1997 ▶) and *DIAMOND* (Brandenburg, 1998 ▶); software used to prepare material for publication: *SHELXL97*.

## Supplementary Material

Crystal structure: contains datablocks global, I. DOI: 10.1107/S1600536810038870/hg2719sup1.cif

Structure factors: contains datablocks I. DOI: 10.1107/S1600536810038870/hg2719Isup2.hkl

Additional supplementary materials:  crystallographic information; 3D view; checkCIF report

## Figures and Tables

**Table 1:** Hydrogen-bond geometry (Å, °)

*D*—H⋯*A*	*D*—H	H⋯*A*	*D*⋯*A*	*D*—H⋯*A*
C9—H9*C*⋯O2^i^	0.98	2.51	3.473 (5)	166
C14—H14⋯O2^ii^	0.95	2.56	3.376 (4)	145

Symmetry codes: (i) 
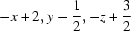
; (ii) 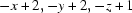.

**Fig. 1.**
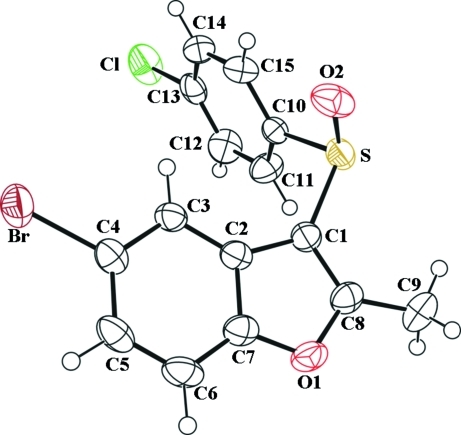
The molecular structure of the title compound with the atom numbering scheme. Displacement ellipsoids are drawn at the 50% probability level. H atoms are presented as a small spheres of arbitrary radius.

**Fig. 2.**
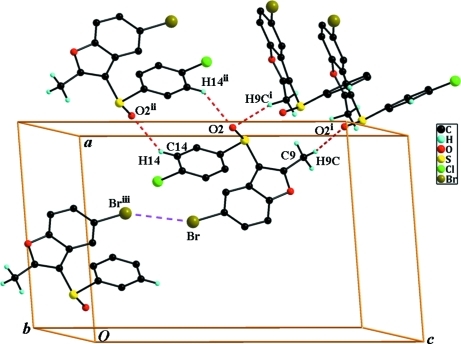
A view of the C—H···O and Br···Br interactions (dotted lines) in the crystal structure of the title compound. [Symmetry codes: (i) - x +2, y - 1/2, - z + 3/2; (ii) - x + 2, - y + 2, - z + 1; (iii) - x + 1, - y + 3, - z + 1.]

## supplementary crystallographic information

### Comment

Many compounds involving a benzofuran skeleton have received much attention owing to their important pharmacological properties such as antifungal, antimicrobial, antitumor and antiviral activities (Aslam *et al.*, 2006; Galal *et al.*, 2009; Khan *et al.*, 2005). These compounds widely occur in nature (Akgul & Anil, 2003; Soekamto *et al.*, 2003). As a part of our study of the substituent effect on the solid state structures of 3-(4-chlorophenylsulfinyl)-2-methyl-1-benzofuran analogues (Choi *et al.*, 2010**a*,b*), we report herein on the crystal structure of the title compound.

In the title molecule (Fig. 1). the benzofuran unit is essentially planar, with a mean deviation of 0.090 (2) Å from the least-squares plane defined by the nine constituent atoms. The 4-chlorophenyl ring makes a dihedral angle of 89.55 (9)° with the mean plane of the benzofuran fragment. The crystal packing (Fig. 2) is stabilized by weak intermolecular C—H···O hydrogen bonds; the first one between a methyl H atom and the oxygen of the S═O unit [C9—H9C···O2^i^; see Table 1], and the second one between the 4-chlorophenyl H atom and the oxygen of the S═O unit [C14—H14···O2^ii^; see Table 1], respectively. The crystal packing (Fig. 2) is further stabilized by an intermolecular a Br—Br^iii^ interaction at 3.783 (3) Å.

### Experimental

77% 3-chloroperoxybenzoic acid (202 mg, 0.9 mmol) was added in small portions to a stirred solution of 5-bromo-3-(4-chlorophenylsulfanyl)-2-methyl-1-benzofuran (289 mg, 0.8 mmol) in dichloromethane (30 mL) at 273 K. After being stirred at room temperature for 5h, the mixture was washed with saturated sodium bicarbonate solution and the organic layer was separated, dried over magnesium sulfate, filtered and concentrated at reduced pressure. The residue was purified by column chromatography (silica gel, hexane–ethyl acetate, 1:1 v/v) to afford the title compound as a colorless solid [yield 83%, m.p. 404–405 K; *R*_f_ = 0.64 (hexane–ethyl acetate, 1:1 v/v)]. Single crystals suitable for X-ray diffraction were prepared by slow evaporation of a solution of the title compound in acetone at room temperature.

### Refinement

All H atoms were positioned geometrically and refined using riding model, with C—H = 0.93 Å for aryl and 0.98 Å for methyl H atoms. *U*_iso_(H) = 1.2*U*_eq_(C) for aryl and 1.5*U*_eq_(C) for methyl H atoms.

### Crystal data

**Table:**

C_15_H_10_BrClO_2_S	*F*(000) = 736
*M**_r_* = 369.65	*D*_x_ = 1.684 Mg m^−^^3^
Monoclinic, *P*2_1_/*c*	Mo *K*α radiation, λ = 0.71073 Å
Hall symbol: -P 2ybc	Cell parameters from 4598 reflections
*a* = 11.530 (6) Å	θ = 2.4–27.8°
*b* = 5.834 (3) Å	µ = 3.14 mm^−^^1^
*c* = 22.045 (13) Å	*T* = 173 K
β = 100.602 (16)°	Block, colourless
*V* = 1457.6 (15) Å^3^	0.20 × 0.16 × 0.15 mm
*Z* = 4	

### Data collection

**Table:**

Bruker SMART APEXII CCD diffractometer	3169 independent reflections
Radiation source: rotating anode	2505 reflections with *I* > 2σ(*I*)
graphite multilayer	*R*_int_ = 0.054
Detector resolution: 10.0 pixels mm^-1^	θ_max_ = 27.0°, θ_min_ = 1.8°
φ and ω scans	*h* = −14→14
Absorption correction: multi-scan (*SADABS*; Bruker, 2009)	*k* = −7→7
*T*_min_ = 0.625, *T*_max_ = 0.746	*l* = −28→28
12344 measured reflections	

### Refinement

**Table:**

Refinement on *F*^2^	Primary atom site location: structure-invariant direct methods
Least-squares matrix: full	Secondary atom site location: difference Fourier map
*R*[*F*^2^ > 2σ(*F*^2^)] = 0.040	Hydrogen site location: difference Fourier map
*wR*(*F*^2^) = 0.125	H-atom parameters constrained
*S* = 1.08	*w* = 1/[σ^2^(*F*_o_^2^) + (0.0713*P*)^2^] where *P* = (*F*_o_^2^ + 2*F*_c_^2^)/3
3169 reflections	(Δ/σ)_max_ = 0.001
182 parameters	Δρ_max_ = 0.37 e Å^−^^3^
0 restraints	Δρ_min_ = −0.61 e Å^−^^3^

### Special details

**Table:**

Geometry. All esds (except the esd in the dihedral angle between two l.s. planes) are estimated using the full covariance matrix. The cell esds are taken into account individually in the estimation of esds in distances, angles and torsion angles; correlations between esds in cell parameters are only used when they are defined by crystal symmetry. An approximate (isotropic) treatment of cell esds is used for estimating esds involving l.s. planes.
Refinement. Refinement of F^2^ against ALL reflections. The weighted R-factor wR and goodness of fit S are based on F^2^, conventional R-factors R are based on F, with F set to zero for negative F^2^. The threshold expression of F^2^ > 2sigma(F^2^) is used only for calculating R-factors(gt) etc. and is not relevant to the choice of reflections for refinement. R-factors based on F^2^ are statistically about twice as large as those based on F, and R- factors based on ALL data will be even larger.

### Fractional atomic coordinates and isotropic or equivalent isotropic displacement parameters (Å^2^)

**Table:**

	*x*	*y*	*z*	*U*_iso_*/*U*_eq_	
Br	0.49545 (3)	1.26359 (6)	0.558213 (16)	0.05190 (16)	
Cl	0.73989 (8)	0.5654 (2)	0.33164 (3)	0.0649 (3)	
S	0.93528 (6)	0.61437 (15)	0.61528 (3)	0.0386 (2)	
O1	0.69728 (19)	0.4763 (4)	0.71682 (8)	0.0423 (5)	
O2	0.9809 (2)	0.8550 (5)	0.62575 (10)	0.0516 (6)	
C1	0.8083 (3)	0.5913 (5)	0.64826 (12)	0.0345 (6)	
C2	0.7048 (3)	0.7367 (5)	0.64045 (12)	0.0328 (6)	
C3	0.6625 (2)	0.9187 (5)	0.60174 (12)	0.0344 (6)	
H3	0.7039	0.9731	0.5711	0.041*	
C4	0.5576 (3)	1.0161 (5)	0.61011 (12)	0.0371 (6)	
C5	0.4941 (3)	0.9380 (6)	0.65489 (13)	0.0433 (7)	
H5	0.4221	1.0104	0.6590	0.052*	
C6	0.5362 (3)	0.7563 (6)	0.69289 (13)	0.0453 (8)	
H6	0.4950	0.7011	0.7235	0.054*	
C7	0.6407 (3)	0.6592 (6)	0.68429 (12)	0.0368 (6)	
C8	0.7994 (3)	0.4395 (5)	0.69429 (12)	0.0371 (7)	
C9	0.8777 (4)	0.2533 (6)	0.72349 (16)	0.0533 (9)	
H9A	0.9466	0.2421	0.7035	0.080*	
H9B	0.8346	0.1077	0.7189	0.080*	
H9C	0.9037	0.2870	0.7674	0.080*	
C10	0.8689 (2)	0.6021 (5)	0.53503 (12)	0.0300 (6)	
C11	0.8105 (3)	0.4075 (5)	0.50999 (14)	0.0451 (8)	
H11	0.7987	0.2828	0.5359	0.054*	
C12	0.7695 (3)	0.3952 (6)	0.44718 (15)	0.0488 (8)	
H12	0.7280	0.2639	0.4293	0.059*	
C13	0.7902 (2)	0.5784 (6)	0.41089 (13)	0.0390 (7)	
C14	0.8486 (3)	0.7708 (5)	0.43509 (15)	0.0434 (8)	
H14	0.8616	0.8944	0.4091	0.052*	
C15	0.8881 (3)	0.7822 (5)	0.49797 (14)	0.0395 (7)	
H15	0.9286	0.9148	0.5157	0.047*	

### Atomic displacement parameters (Å^2^)

**Table:**

	*U*^11^	*U*^22^	*U*^33^	*U*^12^	*U*^13^	*U*^23^
Br	0.0453 (2)	0.0542 (3)	0.0552 (2)	0.01571 (15)	0.00677 (17)	0.00300 (14)
Cl	0.0517 (5)	0.1073 (8)	0.0349 (4)	0.0106 (5)	0.0053 (4)	−0.0076 (4)
S	0.0285 (4)	0.0529 (5)	0.0344 (4)	0.0028 (3)	0.0059 (3)	0.0022 (3)
O1	0.0496 (13)	0.0516 (13)	0.0274 (9)	−0.0036 (11)	0.0116 (9)	0.0057 (8)
O2	0.0441 (13)	0.0659 (15)	0.0450 (12)	−0.0217 (13)	0.0090 (10)	−0.0112 (11)
C1	0.0330 (15)	0.0442 (17)	0.0269 (12)	0.0012 (13)	0.0067 (11)	0.0009 (11)
C2	0.0307 (15)	0.0414 (17)	0.0263 (12)	−0.0052 (12)	0.0054 (11)	−0.0035 (10)
C3	0.0310 (14)	0.0427 (17)	0.0312 (13)	−0.0006 (13)	0.0099 (11)	0.0014 (11)
C4	0.0320 (14)	0.0464 (17)	0.0323 (13)	0.0004 (14)	0.0040 (12)	−0.0075 (12)
C5	0.0300 (15)	0.064 (2)	0.0374 (14)	0.0020 (15)	0.0105 (12)	−0.0127 (14)
C6	0.0412 (18)	0.068 (2)	0.0301 (15)	−0.0078 (15)	0.0159 (14)	−0.0050 (13)
C7	0.0394 (16)	0.0462 (17)	0.0252 (12)	−0.0063 (14)	0.0067 (12)	−0.0033 (12)
C8	0.0436 (16)	0.0414 (16)	0.0257 (12)	−0.0029 (14)	0.0046 (12)	−0.0003 (11)
C9	0.068 (3)	0.051 (2)	0.0385 (16)	0.0065 (16)	0.0032 (17)	0.0119 (13)
C10	0.0256 (13)	0.0325 (14)	0.0334 (13)	0.0047 (11)	0.0096 (11)	0.0010 (10)
C11	0.054 (2)	0.0348 (16)	0.0470 (16)	−0.0054 (15)	0.0095 (15)	0.0040 (13)
C12	0.052 (2)	0.0419 (18)	0.0496 (17)	−0.0068 (16)	0.0025 (15)	−0.0083 (14)
C13	0.0278 (14)	0.058 (2)	0.0336 (14)	0.0102 (14)	0.0110 (12)	−0.0027 (13)
C14	0.0409 (18)	0.052 (2)	0.0395 (15)	0.0005 (15)	0.0137 (14)	0.0109 (13)
C15	0.0374 (17)	0.0410 (17)	0.0424 (15)	−0.0088 (13)	0.0132 (14)	−0.0017 (12)

### Geometric parameters (Å, °)

**Table:**

Br—C4	1.899 (3)	C6—C7	1.376 (5)
Br—Br^i^	3.7826 (17)	C6—H6	0.9500
Cl—C13	1.738 (3)	C8—C9	1.482 (4)
S—O2	1.502 (3)	C9—H9A	0.9800
S—C1	1.756 (3)	C9—H9B	0.9800
S—C10	1.795 (3)	C9—H9C	0.9800
O1—C8	1.376 (4)	C10—C15	1.373 (4)
O1—C7	1.381 (4)	C10—C11	1.382 (4)
C1—C8	1.365 (4)	C11—C12	1.380 (4)
C1—C2	1.448 (4)	C11—H11	0.9500
C2—C3	1.393 (4)	C12—C13	1.382 (5)
C2—C7	1.395 (4)	C12—H12	0.9500
C3—C4	1.379 (4)	C13—C14	1.366 (5)
C3—H3	0.9500	C14—C15	1.379 (4)
C4—C5	1.409 (4)	C14—H14	0.9500
C5—C6	1.383 (5)	C15—H15	0.9500
C5—H5	0.9500		
			
C4—Br—Br^i^	155.35 (9)	C1—C8—C9	132.5 (3)
O2—S—C1	107.65 (14)	O1—C8—C9	116.8 (3)
O2—S—C10	105.22 (13)	C8—C9—H9A	109.5
C1—S—C10	99.69 (13)	C8—C9—H9B	109.5
C8—O1—C7	106.7 (2)	H9A—C9—H9B	109.5
C8—C1—C2	107.2 (3)	C8—C9—H9C	109.5
C8—C1—S	122.7 (2)	H9A—C9—H9C	109.5
C2—C1—S	129.6 (2)	H9B—C9—H9C	109.5
C3—C2—C7	120.1 (3)	C15—C10—C11	120.7 (3)
C3—C2—C1	135.0 (3)	C15—C10—S	118.0 (2)
C7—C2—C1	104.9 (2)	C11—C10—S	120.9 (2)
C4—C3—C2	116.6 (3)	C12—C11—C10	119.7 (3)
C4—C3—H3	121.7	C12—C11—H11	120.2
C2—C3—H3	121.7	C10—C11—H11	120.2
C3—C4—C5	122.9 (3)	C11—C12—C13	118.5 (3)
C3—C4—Br	118.4 (2)	C11—C12—H12	120.8
C5—C4—Br	118.7 (2)	C13—C12—H12	120.8
C6—C5—C4	120.1 (3)	C14—C13—C12	122.3 (3)
C6—C5—H5	119.9	C14—C13—Cl	118.5 (2)
C4—C5—H5	119.9	C12—C13—Cl	119.1 (2)
C7—C6—C5	116.8 (3)	C13—C14—C15	118.7 (3)
C7—C6—H6	121.6	C13—C14—H14	120.7
C5—C6—H6	121.6	C15—C14—H14	120.7
C6—C7—O1	126.1 (3)	C10—C15—C14	120.1 (3)
C6—C7—C2	123.4 (3)	C10—C15—H15	120.0
O1—C7—C2	110.5 (3)	C14—C15—H15	120.0
C1—C8—O1	110.7 (3)		
			
O2—S—C1—C8	−120.0 (3)	C1—C2—C7—O1	−1.0 (3)
C10—S—C1—C8	130.5 (2)	C2—C1—C8—O1	0.0 (3)
O2—S—C1—C2	50.7 (3)	S—C1—C8—O1	172.54 (19)
C10—S—C1—C2	−58.8 (3)	C2—C1—C8—C9	−178.9 (3)
C8—C1—C2—C3	−179.5 (3)	S—C1—C8—C9	−6.4 (5)
S—C1—C2—C3	8.7 (5)	C7—O1—C8—C1	−0.7 (3)
C8—C1—C2—C7	0.6 (3)	C7—O1—C8—C9	178.4 (3)
S—C1—C2—C7	−171.2 (2)	O2—S—C10—C15	10.8 (3)
C7—C2—C3—C4	1.0 (4)	C1—S—C10—C15	122.2 (2)
C1—C2—C3—C4	−178.9 (3)	O2—S—C10—C11	−175.8 (2)
C2—C3—C4—C5	−0.4 (4)	C1—S—C10—C11	−64.3 (3)
C2—C3—C4—Br	−179.6 (2)	C15—C10—C11—C12	−0.9 (5)
C3—C4—C5—C6	0.0 (5)	S—C10—C11—C12	−174.2 (3)
Br—C4—C5—C6	179.2 (2)	C10—C11—C12—C13	1.1 (5)
C4—C5—C6—C7	−0.2 (4)	C11—C12—C13—C14	−0.7 (5)
C5—C6—C7—O1	−179.5 (3)	C11—C12—C13—Cl	179.7 (3)
C5—C6—C7—C2	0.8 (5)	C12—C13—C14—C15	0.0 (5)
C8—O1—C7—C6	−178.6 (3)	Cl—C13—C14—C15	179.7 (3)
C8—O1—C7—C2	1.1 (3)	C11—C10—C15—C14	0.3 (5)
C3—C2—C7—C6	−1.3 (4)	S—C10—C15—C14	173.8 (3)
C1—C2—C7—C6	178.7 (3)	C13—C14—C15—C10	0.2 (5)
C3—C2—C7—O1	179.0 (2)		

Symmetry codes: (i) −*x*+1, −*y*+3, −*z*+1.

### Hydrogen-bond geometry (Å, °)

**Table:**

*D*—H···*A*	*D*—H	H···*A*	*D*···*A*	*D*—H···*A*
C9—H9C···O2^ii^	0.98	2.51	3.473 (5)	166
C14—H14···O2^iii^	0.95	2.56	3.376 (4)	145

Symmetry codes: (ii) −*x*+2, *y*−1/2, −*z*+3/2; (iii) −*x*+2, −*y*+2, −*z*+1.
